# The Multifaceted Roles of Copper in Cancer: A Trace Metal Element with Dysregulated Metabolism, but Also a Target or a Bullet for Therapy

**DOI:** 10.3390/cancers12123594

**Published:** 2020-12-01

**Authors:** Pierre Lelièvre, Lucie Sancey, Jean-Luc Coll, Aurélien Deniaud, Benoit Busser

**Affiliations:** 1 Institute for Advanced Biosciences, UGA INSERM U1209 CNRS UMR5309, 38700 La Tronche, France; pierre.lelievre@univ-grenoble-alpes.fr (P.L.); lucie.sancey@univ-grenoble-alpes.fr (L.S.); jean-luc.coll@univ-grenoble-alpes.fr (J.-L.C.); 2 Univ. Grenoble Alpes, CNRS, CEA, IRIG, Laboratoire de Chimie et Biologie des Métaux, 38000 Grenoble, France; 3 Department of Clinical Biochemistry, Grenoble Alpes University Hospital, 38043 Grenoble, France

**Keywords:** copper homeostasis, cancer, prognostic, diagnostic, therapy

## Abstract

**Simple Summary:**

Copper is an essential element for human life. However, its redox activity can be detrimental for the cell that developed highly coordinated pathways to chelate and traffic copper through the cell or the organism. Owing to its important role in functions essential for cell growth and metabolism, copper concentrations are frequently dysregulated in tumors. In this review, we describe normal and cancer-altered copper homeostasis mechanisms. Moreover, on the basis of this knowledge, we expose not only copper-related diagnostic and prognostic markers for oncology but also therapeutic strategies to act on copper homeostasis to fight against cancer.

**Abstract:**

In the human body, copper (Cu) is a major and essential player in a large number of cellular mechanisms and signaling pathways. The involvement of Cu in oxidation–reduction reactions requires close regulation of copper metabolism in order to avoid toxic effects. In many types of cancer, variations in copper protein levels have been demonstrated. These variations result in increased concentrations of intratumoral Cu and alterations in the systemic distribution of copper. Such alterations in Cu homeostasis may promote tumor growth or invasiveness or may even confer resistance to treatments. Once characterized, the dysregulated Cu metabolism is pinpointing several promising biomarkers for clinical use with prognostic or predictive capabilities. The altered Cu metabolism in cancer cells and the different responses of tumor cells to Cu are strongly supporting the development of treatments to disrupt, deplete, or increase Cu levels in tumors. The metallic nature of Cu as a chemical element is key for the development of anticancer agents via the synthesis of nanoparticles or copper-based complexes with antineoplastic properties for therapy. Finally, some of these new therapeutic strategies such as chelators or ionophores have shown promising results in a preclinical setting, and others are already in the clinic.

## 1. Introduction

Trace elements such as copper (Cu) are involved in many physiological processes. It has been shown that disturbances in copper homeostasis lead to structural abnormalities or loss of certain essential physiological functions. It has been clearly demonstrated that copper homeostasis is deregulated in many cancers. In addition, numerous studies showed that the deregulation of trace element homeostasis might be, at the same time, the cause and consequence of carcinogenesis. Some studies have also revealed that these dysregulations could be of clinical interest as a prognostic and/or predictive biomarker of a response to treatment. Accordingly, several therapeutic strategies targeting or using trace elements have been developed. In view of such rich literature, we present the most significant studies on cell mechanisms relating to Cu homeostasis dysregulation and cancer. This review is also an opportunity to present the discrepant results on this subject. Finally, in this work, we review the main therapeutic strategies targeting Cu or using Cu as a central player for cancer treatment.

## 2. Copper Normal Metabolism

Cu is an essential trace element with a short half-life of about a month. The required daily intake of Cu is 0.8 mg [1]. Cu concentrations fluctuate from 1 to 10 mg/g of tissues, and the Cu concentration in blood plasma is approximately 1000 ng/mL. However, Cu concentration may vary depending on various factors [2].

In the body, copper is present in two redox states, namely, Cu(I) and Cu(II). In fluids, Cu is in its Cu(II) state, and in the intracellular reducing environment, it is mainly found in its Cu(I) state. However, in redox enzymes, Cu is shuttling between Cu(I) and Cu(II). Cu plays an important role in various cellular functions. A list of 54 Cu-binding proteins was established using a bioinformatic approach (Table 1) [3]. However, this list is not exhaustive because of the fact that other copper-dependent proteins and/or proteins involved in copper metabolism may be discovered in the future. Cu is also involved in angiogenesis cellular mechanisms and in other signaling pathways [4,5,6].

**Figure 1:**
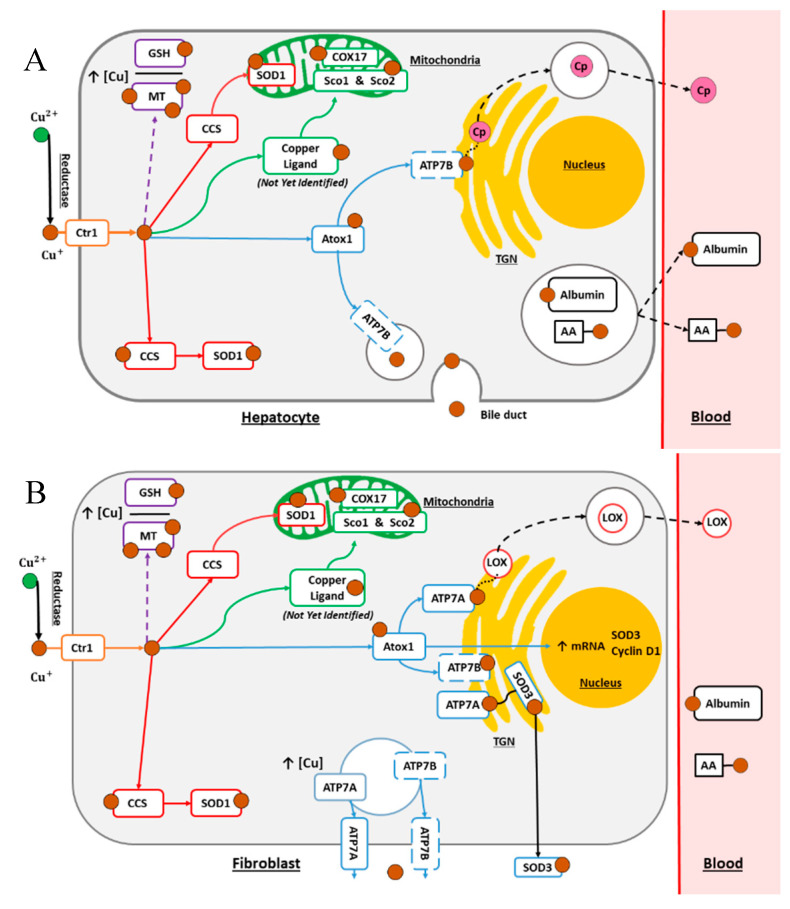
Cellular copper homeostasis in hepatocytes (**A**) and fibroblasts (**B**). In cells, the internalization of reduced Cu occurs via Ctr1. Then, Cu is involved in the control of oxidative stress (red arrows) through a copper chaperone for superoxide dismutase protein (CCS) and cytoplasmic Cu/Zn superoxide dismutase 1 (SOD1). Copper can be stored in its main storage reservoir, the mitochondria, where the proteins COX17, Sco1, and Sco2 play a role in its incorporation into complex IV of the respiratory chain (green arrows). Nevertheless, in the mitochondrial pathway, the identity of the chaperone that carries Cu across the mitochondrial membranes is not clearly identified. The excess Cu can also be sequestered by metallothioneins (MT) and/or bound by glutathione (GSH; dashed purple arrows). (**A**) In hepatocytes, copper can be secreted via the bile duct or released in the blood stream bound to ceruloplasmin (Cp) after transport by Atox1 and ATP7B proteins (blue arrows) and transit in the trans-Golgi network (TGN). Cu is released into the blood stream and travels by binding to Cp, amino acids (AA), or albumin. (**B**) In fibroblasts, ATP7A carries Cu from Atox1 to the TGN for its integration into lysyl oxidase (LOX) or extracellular superoxide dismutase 3 (SOD3) before their extracellular release. In addition, the excess intracellular Cu can be removed from the cell through ATP7A and ATP7B.

Although essential for normal cell function, free Cu can induce toxicity to cells. In physiological conditions, Cu is always bound to peptides or proteins to prevent uncontrolled redox activity. Several cellular mechanisms allow the precise regulation of the spatial and temporal distributions of Cu (Figure 1A,B). In human cells, Cu is internalized by copper transport protein 1 (Ctr1) [7]. Additionally, a second Cu transport protein Ctr2 may be involved in copper transport, but it has a lower affinity for Cu, and its role remains unclear [8]. The Ctr1 protein is trafficking between the plasma membrane and intracellular vesicles to control the entry of Cu into the cell. Thus, in the case of increased copper levels, Ctr1 is internalized in intracellular vesicles. The Ctr1 protein is essential for the stability of Ctr2, and Ctr1 and Ctr2 have interconnected functions in Cu homeostasis [9]. Finally, a model suggests that the regulation of systemic copper and intracellular mobilization involves the cleavage of the ecto-domain of Ctr1 that binds copper. Ctr2 may regulate the formation of the truncated form of Ctr1 (tCtr1) [10]. Moreover, the absence of Ctr2 leads to the accumulation of Cu in the endosomal compartments. Once in the cytoplasm, Cu is distributed through four main pathways (Figure 1A,B).

### 2.1. The Secretory Pathway

The cytoplasmic Cu chaperone, antioxidant-1 protein (Atox1), brings Cu to copper transport ATPases, namely, ATP7A and ATP7B. Both are present at the trans-Golgi network level, importing Cu into the Golgi apparatus lumen for the maturation of Cu-dependent target enzymes such as ceruloplasmin (Cp), lysyl oxidase (LOX), tyrosinase, or extracellular Cu/Zn superoxide dismutase 3 (SOD3), which are all secreted by exocytosis. Concerning SOD3, it has been shown that the Cu acquisition pathway for SOD3 involves at the same times the Atox1 chaperone and copper transporter ATP7A in the trans-Golgi network [6]. Moreover, it turns out that Atox1 has a dual function, and it is a copper-dependent transcription factor for SOD3 and cyclin D1 [11]. In this Cu transport pathway, it was also shown that vascular ATP7A is necessary for the complete activation of SOD3 via a Cu-dependent interaction [6]. Cu bound to Cp is the main form of Cu in the blood (80–90%). Cp is mainly synthetized in hepatocytes and in the kidneys, placenta, breast, or brain. Macrophages and mononuclear cells from the blood can also produce Cp during inflammation [2]. The LOX enzyme is synthesized into an inactive proenzyme that goes through a proteolytic treatment to obtain a mature enzyme and propeptide [12,13]. This enzyme ensures the reticulation of collagen and elastin to preserve the rigidity and structural stability of the extracellular matrix. Tyrosinase is a copper-containing monooxygenase, a major enzyme involved in the critical stage of the melanin pigment biosynthesis pathway [14].

As soon as intracellular Cu concentration increases and is in excess, both ATP7A/B proteins are relocated to the plasma membrane to excrete Cu out of the cell [7]. The expression levels of ATP7A and ATP7B vary depending on the tissue. The ATP7A protein (Menkes’ protein) is expressed in all cell types, except hepatocytes. The ATP7B protein (Wilson’s protein) is mainly expressed in the liver and in the kidney, placenta, heart, brain, and lung tissues [15]. ATP7B is central for Cu homeostasis in the liver and thereof at the organismal level because the liver regulates the Cu concentrations in the blood. Indeed, this protein ensures the excretion of copper from hepatocytes to the bile in response to Cu overload, which is the main process to decrease Cu level in an organism. In addition, in plasma or other fluids, Cu is bound to different amino acids, peptides, or proteins such as histidine, albumin, transcuprein, or metallothioneins [16,17,18,19]. The secretory pathway can be subject to disruptions due to mutations in the genes coding for the proteins ATP7A or ATP7B, leading to Wilson’s and Menkes diseases, respectively. The former is an inherited autosomal recessive genetic disease resulting in copper accumulation in the body, principally in the liver and brain, and triggering hepatic and neuropsychiatric symptoms [20]. Menkes disease is an X-linked recessive disorder in which patients lack Cu, which induces growth delays and nervous system alteration

### 2.2. The Cytosolic Pathway

The copper chaperone for superoxide dismutase (CCS) protein transports Cu toward intracellular dimeric Cu/Zn superoxide dismutase 1 (SOD1) in the cytosol and mitochondrial intermembrane space (IMS) [6] (Figure 1A,B). The proteins of the SOD family are key players in the defense against oxidative stress because it catalyzes the degradation of superoxide anions into hydrogen peroxide and oxygen [6]. Several isoforms of SOD exist including SOD2, which is a mitochondrial manganese (Mn) containing enzyme (MnSOD) and two others, which contain Cu. These two other enzymes are the intracellular dimeric Cu/Zn-SOD (SOD1) and extracellular tetrameric Cu/Zn superoxide dismutase 3 (SOD3) as seen previously.

In the cytosol, Cu can be directly bound to a multitude of kinases. We can cite the interaction between copper and the kinases of the serine/threonine-protein kinase B-raf (BRAF) signaling pathway being dual specificity mitogen-activated protein kinase kinase mek-1/2 (MEK1/2) [21]. Recently, another metal–kinase interaction has been demonstrated. Moreover, Cu is necessary for the activity of the autophagic serine/threonine-protein kinase ULK1 and serine/threonine-protein kinase ULK2 (ULK1/2) via a direct Cu–ULK1/2 linkage [22].

To conclude, copper (Cu) is able to bind to various cytosolic proteins such as growth factors, cell signaling proteins, or structural proteins [23]. Within the cell, Cu can directly regulate the activity of these protein partners such as kinases. Therefore, many signaling pathways are copper dependent.

### 2.3. The Mitochondrial Pathway

Copper is an important trace element in the physiology of the mitochondria, which also have an essential role in copper homeostasis [24]. Cu is actively transported to the mitochondrial matrix, where it is required to mature in a first instance cytochrome c oxidase (COX), which is complex IV of the respiratory chain. The assembly of COX requires different subunits and cofactors added in a sequential and orderly process [25]. The identity of the chaperone driving Cu through the mitochondrial membranes is not clearly established, but it could be either COX17 or a non-protein metallophore of unknown identity, and it is named Cu ligand (Figure 1A,B) [26]. Moreover, recent research conducted by Cobine et al. has shown that the mammalian phosphate carrier SLC25A3 is a mitochondrial copper transporter necessary for the biogenesis of cytochrome c oxidase [27]. Within the mitochondria, the metallochaperones synthesis of cytochrome c oxidase 1 (Sco1) and synthesis of cytochrome c oxidase 2 (Sco2; Figure 1A,B) are then involved in further Cu insertion into the COX. Being the main intracellular Cu storage reservoir, the mitochondria host a Cu-dependent energy production via oxidative phosphorylation [7,28].

### 2.4. Cellular Processes for Metal Detoxification

In cells, Cu is also stored in lysosomes [29,30]. Metallo-reductases, such as the human 6-transmembrane epithelial antigen of prostate (STEAP) family proteins, are necessary to maintain copper in its Cu(I) state because lysosomes are an oxidative environment. These reductases, principally STEAP3 and STEAP4, are located in intracellular vesicles and are involved in many biological processes, such as the regulation of cell proliferation and apoptosis [31,32].

Metallothioneins (MTs) and glutathione (GSH) are other important players for intracellular storage and sequestration of excess Cu. Metallothioneins are cysteine-rich cytosplasmic proteins. They play an important role in the homeostasis and detoxification of metals. They have the ability to bind to metals such as Cu and zinc (Zn). In humans, there are four distinct MTs, identified as MT1, MT2, MT3, and MT4. MT1 and MT2 are the main ones, inducible via numerous stimuli. The MT3 and MT4 proteins are mainly expressed in the central nervous system [33]. The GSH is involved not only in several mechanisms such as xenobiotics metabolism and redox signaling but also in the transfer and detoxification of metal ions such as Cu [34,35]. The majority of cytosolic copper is found in Cu(I)–GSH complexes. Indeed, this complex is considered a major contributor to the exchangeable pool of Cu in the cytosol. However, this point is subject to debate in the scientific community [36,37].

## 3. Copper Metabolism in Cancers

When compared with nonpathological conditions, variations in Cu concentrations or in the Cu/Zn ratios were associated with many cancers. The Cu/Zn ratio is of clinical importance because of its relationship with aging, nutritional status, oxidative stress, inflammation, and immune abnormalities [38,39]. Increased Cu levels were associated with decreased Zn levels in a meta-analysis in bladder cancer [40] and in breast cancer, colorectal cancer (CRC), and prostate cancers [41,42,43,44,45,46,47]. Importantly, some discrepant studies reported decreases in Cu levels in CRC and breast cancers [48,49].

Cu is important for functions involved in proliferation or angiogenesis, which are central for tumorigenesis and cancer development. Copper is acting on different molecular pathways leading to a proangiogenic response necessary for carcinogenesis processes. It appears that copper also influences the spread and formation of secondary tumors via the activation of enzymes responsible for cell proliferation. It is therefore not surprising that Cu concentration is increased in tumor areas [50,51,52]. More recently, it was shown that specific Cu accumulation can be observed in cancer cells themselves [51,53]. It is worth noting that the accumulation of Cu in the nuclear region has been found in breast cancer cells [54].

Moreover, early reports described the increases of serum Cu in cancer patients, sometimes even correlated with the grade of the cancer [55]. High serum Cu levels were also found in cancer patients resistant to chemotherapy compared to patients responding to treatment [55]. However, this remains unexplained up to now, and several data on different types of cancer where published, sometimes being contradictory.

More recently, isotopic fractionation was developed for biological samples, usually measured in blood. It has been shown that the isotopic ^63^Cu/^65^Cu ratio is modified in the serum of cancer patients [56], where the lighter isotope is enriched in the blood. This phenomenon could be due to metabolism modifications in cancers such as increased glycolysis, and therefore higher lactate production. This would explain the higher excretion of ^63^Cu by ATP7A in the blood stream. Moreover, it has been shown that the Cu isotopic ratio can be used as an early diagnostic biomarker for cancer, usable several months before other classical protein markers. Since Cu turnover is short (i.e., about one month), it is also convenient as a follow-up marker during treatment to monitor therapeutic efficacy.

Altogether, it is clear that Cu is central for cancer development at each step from tumorigenesis to metastasis. Cancer cell metabolism is also affecting Cu metabolism. Therefore, it is expected that prognostic and diagnostic markers for cancer can be identified in relation with Cu.

## 4. The Use of Copper Proteins as Cancer Biomarkers

As discussed earlier, it is now widely accepted that disruption of copper homeostasis occurs in several cases of cancer. This can be linked with increases or decreases in protein levels. Large-scale studies such as the analysis of TCGA data in breast cancer revealed an increase in several copper-related proteins, including ATP7B and Ctr1 [3]. Moreover, Ctr1, Sco1, and COX11 were increased in a transcriptomic analysis of copper homeostasis genes in CRC samples [57]. Consequently, copper homeostasis proteins appear to be promising candidates as predictive biomarkers of the treatment response or as prognostic biomarkers, and their clinical use may guide the choice for optimal therapeutic strategy in the future (Table 2).

### 4.1. Involvement of Copper Metabolism Proteins in Metastasis Formation

Studies have demonstrated that the deregulation of certain copper metabolism proteins has an impact on the migration of cells and on the formation of metastasis.

The Atox1 protein is increased in many types of cancer tissues [3]. In blood, breast, and skin cancers, the mRNA levels of Atox1 are significantly higher compared to those in nonpathological tissues (Table 1) [58]. Moreover, it has been shown that Atox1 has a role in the migration of cancer cells in breast cancer [127]. This protein also promotes inflammatory neovascularization through its putative action as a transcription factor and as a cytoplasmic Cu chaperone [4]. Atox1 may be a potential prognostic biomarker for estrogen-receptor (ER)-positive and early stages of breast cancers because increased expression levels of Atox1 correlated with poor survival for stages 1 and 2 breast cancers. However, preclinical studies and clinical trials are required to better understand the carcinogenic role of Atox1 [128]. In melanoma, a correlation between the overexpression of Atox1 and poor prognosis was observed because the knockdown of Atox1 decreased cell growth and BRAF V600E-dependent signaling in human melanoma cell lines [58]. From a tissue microarray, the increased nuclear translocation of Atox1 was observed in metastatic CRC and was correlated with the severity of the disease [59]. The nuclear localization of Atox1 was also correlated with increased migration capabilities in breast cancer cells in an ATP7A- and LOX-dependent mechanism [129]. Indeed, the colocalization of the three proteins, namely, Atox1, ATP7A, and LOX, was observed at the lamellipodia border of the cell. The reason is unclear, but it could be a way to maximize LOX maturation close to its excretion site, where it plays a major role in cancer cell migration and metastasis. Concerning the ATP7A protein, it is also deregulated in many cancers, such as in pancreatic cancer, where ATP7A is upregulated compared to that in chronic pancreatitis [60]. The ATP7A protein plays an important role in the formation of metastases in breast cancer and induces the migration of vascular smooth muscle cells [130]. In addition, high levels of ATP7A expression in primary tumors are associated with reduced survival according to publicly available databases [131].

Finally, the family of copper-dependent LOX metalloenzymes has a role in tumor metastasis and fibrotic diseases. In the formation of premetastatic niches, cancer cells secrete the LOX protein to stimulate collagen cross-linking and fibronectin synthesis [132]. This secretion promotes the migration and adhesion of tumor cells [133,134]. LOX promotes tumor cell migration and adhesion by activating the focal adhesion kinase (FAK1) [133]. To date, several dysregulations of enzymes from the LOX family were reported in breast, colorectal, prostate, gastric, hepatic, pancreatic, head, and neck cancers, and in skin cancers, including melanoma [74,75,135]. In bladder cancer, LOXL1 and LOXL4 inhibited oncogenic RAS-mediated activation of extracellular signal-regulated kinase (ERK), a tumor suppressive action leading to reduced colony formation [87]. Increased LOX levels may be associated with poor prognosis in different cancers, especially in patients with ER-negative breast cancers [136]. LOX proteins may play complex and paradoxical roles in the metastatic process or may act as tumor suppressors [135]. The mechanism by which copper is supplied to copper-dependent metalloenzymes LOX and LOX-likes (LOXL) is still not well known. A recent study in mice by Shanbhag, V. et al. has shown that the copper transporter ATP7A is necessary and sufficient for the activity of the enzymes LOX and LOXL. In addition, the silencing of ATP7A suppresses carcinogenesis via the loss of several mechanisms such as the loss of focal adhesion kinase (FAK1) phosphorylation and an attenuation of myeloid cell recruitment in the mouse lung. These observations open up opportunities for the development of new therapeutic strategies that could target ATP7A in order to act on the metalloenzymes of the LOX family responsible for metastasis formation [137].

### 4.2. Role of Copper Metabolism in Drug Resistance

Afterward, it was clearly established that certain proteins responsible for copper homeostasis also play a role in drug resistance and more specifically in resistance to platinum salt treatments.

In CRCs, an increase in the level of ATP7A mRNA was also found [57], and ATP7A may be a promising predictive biomarker of drug resistance in human CRCs [63]. In lung cancers, ATP7A protein is only expressed in 40% of tumor tissues, and patients expressing ATP7A had a poorer response to platinum-based chemotherapy. At the cellular level, both mRNA and protein levels of ATP7A were significantly higher in multidrug resistant A549 human lung adenocarcinoma cell lines when compared with the parental sensitive cells. ATP7A expression may thus be a predictive biomarker of chemoresistance and a negative prognostic factor for survival in non-small cell lung cancer (NSCLC) and ovarian cancer patients treated with platinum-based chemotherapy [62,64,65].

The ATP7B protein is also deregulated in cancers. In CRCs, low mRNA or protein levels of ATP7B are associated with a response to oxaliplatin/5-FU treatment in patients [138]. ATP7B was also a predictive biomarker of platinum-based drug resistance in a NSCLC xenograft model [139] and in the clinical setting for CRCs [138], oral squamous carcinomas [69], and esophageal [66] and ovarian [140] carcinomas. In all of these clinical studies, ATP7B was not detected in the adjacent nonneoplastic tissues [66,69,138,140]. In addition, resistance to cisplatin was correlated with increased levels of GSH in ovarian tumor cell lines [141]. In various cancer types, the fluctuations of the total intracellular GSH level could explain the resistance/sensitivity patterns to cisplatin [142].

Ctr1 is the major Cu influx carrier in human cells. Variations in Ctr1 expression were observed both in nonpathological tissues and in various cancer tissues. However, the absence of Ctr1 expression was also reported in some cancers such as cervical squamous carcinoma, prostate carcinoma, and gastric carcinoma [143]. The Ctr1 transporter is also responsible for the absorption of cisplatin, and Ctr1 is logically a key player in resistance and sensitivity mechanisms to platinum-based cancer therapies [144]. Other studies have shown a correlation between increased levels of Ctr1 and better absorption of platinum-based drugs in sensitive cells [109,145,146]. In addition, Ctr1 overexpression resulted in prolonged progression-free survival (PFS) and improved overall survival in patients with stage III NSCLC [107]. Many studies have hypothesized that the development of resistance to platinum-based drugs may be due to the defect in the glycosylation of the Ctr1 protein [147].

Finally, dysregulations of various Cu metabolic proteins can be the cause of resistance to platinum-based anticancer therapies [148]. These deregulations can be positive or negative depending on whether the protein is involved in the influx or efflux of Cu, respectively. Consequently, the drug’s ability to penetrate cells decreases [149]. This is due to the fact that platinum-based drugs use copper metabolism proteins to enter cells via Ctr1 initially [150], and subsequently bind to Atox1, which is responsible for intracellular copper trafficking [151,152]. Moreover, therapeutic platinum salts can be excreted outside the cells by the Cu transporters ATP7A or ATP7B [153,154]. This could explain the downregulation of Ctr1 to limit platinum cellular entry and the subsequent upregulation of ATP7A, ATP7B, and/or Atox1 to induce platinum excretion.

Moreover, copper consumption by growing cancer cells increases. Thus, it uses copper efflux proteins such as ATP7A/B to limit its potential toxicity and regulates its availability for oncogenic enzymes such as LOX and LOX-like proteins [155]. Numerous studies have concluded that the acquisition of resistance to platinum-based drugs is accompanied by an alteration in the absorption and efflux of copper [156,157,158]. These observations are not surprising because the platinum salt drugs interact with copper metabolism proteins [148]. To circumvent these resistance mechanisms against platinum complexes, drug screenings were performed to identify molecules able to maintain Cu transport to the Golgi apparatus while inhibiting platinum salt excretion [159].

### 4.3. Implication of Copper Homeostasis Protein in the Proliferation and Growth of Cancer Cells

Eventually, it turns out that the deregulation of copper metabolism has a role in cell proliferation and growth. We have previously seen that CCS delivers Cu to Cu/Zn superoxide dismutase (SOD1). This Cu is mandatory for its maturation and its role in the control of reactive oxygen species production. Moreover, CCS could have the ability to promote carcinogenesis. The use of a specific inhibitor of CCS and Atox1 has been shown to reduce cancer cell proliferation and tumor growth. However, the potential presence of other targets that might work in synergy should not be excluded [160]. In patients with breast cancer, CCS protein level increases. The ability of CCS to promote proliferation may involve the MAPK/ERK pathway [161]. The expressions of CCS and COX17 are generally higher in lung cancer compared to healthy tissue. However, the expression of COX17 can vary between tumors and cell lines [162]. Another study showed that the upregulation of COX17 function and increased COX activity are also frequent features in lung carcinogenesis [125]. Overexpression of COX17 might also play a significant role in the oncogenesis of lung cancer. Moreover, in studies on human papillomavirus-positive head and neck squamous cell carcinomas, it has been observed that COX16 and COX17 expression was significantly correlated with the survival of the patient [126]. These observations suggest that the deregulation at the level of subunits and/or cofactors necessary for the assembly of cytochrome c oxidase could favor carcinogenesis.

Subsequently, studies have also shown deregulations of proteins of the superoxide dismutase family in some cancers [163]. These deregulations mainly result in decreased levels of SOD3 expression, and they are often associated with genetic alteration [163]. In breast cancer, the decrease in SOD3 mRNA expression levels is synonymous of poor prognosis [117,118]. In addition, studies have shown that overexpression of SOD3 can inhibit proliferation in vitro in various cancers [163]. Lastly, in cases of renal carcinomas, high SOD3 activity led to significant apoptosis [164]. These observations suggest that decreased mRNA levels of SOD confer a selective advantage to cancer cells. The majority of the studies have shown that this protein has a tumor suppressor role in many cancers [165]. Therefore, the deregulation of superoxide dismutase is an important player in oncogenesis.

Finally, recent studies have shown that autophagy has an important role in carcinogenesis. Autophagy is a cell degradation process that has an essential role in the development and differentiation of cells. Autophagy also constitutes a means of confronting intracellular and environmental stress, thus promoting tumor progression [166]. Recent research on autophagy has shown that Cu enhances the survival of cancer cells via the activation of autophagy. Indeed, Cu directly modulates the activity of the autophagic kinases ULK1 and ULK2. They described that a direct interaction between Cu and ULK1 is necessary and sufficient for autophagy signaling and induction [22]. Several studies have revealed the role of Cu in the induction of autophagy and the implication of this mechanism in carcinogenesis [22,167]. Consequently, these observations open up new therapeutic perspectives. To finish on cancer proliferation, correlations between GSH levels and cancer cell growth were found in melanoma and liver cancer [113,168]. In clear cell renal cell carcinoma, tumor progression and metastasis were linked to increased metabolic activity in both GSH and cysteine/methionine metabolism pathways [169].

### 4.4. Deregulation of Other Copper-Dependent Proteins in Cancers

In this section, we saw that the deregulations of homeostasis are multiple and these deregulations affect different stages of carcinogenesis. Nevertheless, several copper-dependent proteins are also affected by deregulation in the context of carcinogenesis, such as tyrosinase, whose expression is increased in melanoma [170].

Other dysregulations of copper-dependent proteins such as coagulation factors V and VIII have been found in different cancers [171,172,173,174]. Coagulation disorders have also been shown to occur in cancer patients. These disorders result in the constant activation of the coagulation system in the blood and malignant effusions [175]. However, it is important to note that no link between coagulation disorders and copper homeostasis dysregulation in cancers has been clearly demonstrated.

To conclude, copper metabolism proteins and copper-dependent proteins are subject to numerous deregulations that can promote tumorigenesis. Therefore, these deregulations represent potential targets in the design of therapeutic strategies.

In the next part of this review, we will present different therapeutic strategies and lines of research based on the therapeutic use or the targeting of Cu and/or Cu–proteins for cancer treatment.

## 5. Copper as a Target or Bullet for Cancer Treatment

### 5.1. Copper Chelation-Based Treatment Strategies

The use of chelators or ionophores is a frequent strategy to target Cu levels in cells [176]. Chelators directly bind and sequester metal ions, whereas ionophores cross cellular membranes in a Cu-bound form and release Cu on the other side of the membrane, generally leading to the increase of the intracellular concentrations of metal ions [177].

The first Cu chelators were developed in the mid-20th century for treating patients with Wilson’s disease, notably D-penicillamine and trientine, which are acting extracellularly. More recently, Cu(I) chelators such as tetrathiomolybdate (TTM) have been developed to act inside cells in a more efficient way [21].

It has been shown that D-penicillamine induces inhibition of human endothelial cell proliferation in vitro and neovascularization in vivo [178]. Afterward, trientine also showed an antineoplastic effect and caused important suppression of tumor development in murine and human hepatocellular carcinoma cell lines [179,180]. Trientine is considered to have a reduced Cu chelating capacity compared to D-penicillamine, but it has a more tolerable toxicity profile.

In fact, the availability of cellular Cu is critical for the activity of MEK1 and MEK2 kinases in the RAS/MAPK signaling pathway. Copper intake promotes the phosphorylation of the MEK1 protein and ERK1 and ERK2 through a Cu–MEK1 interaction [181]. The activation of the copper-dependent mitogen-activated kinase (MAP) pathway is thus a key player in the promotion of tumor growth, and targeting Cu was proven to be a relevant strategy against cancer progression. In a cornerstone study, Brady et al. demonstrated the link between cancer mutational status and variations in cytosolic Cu content in melanoma [21]. The targeting of Cu with TTM induced antitumor effects in cells with BRAF V600E kinase mutations, which gave a strong rationale for the further development of several secondary studies aiming at disrupting the central role of Cu in other BRAF V600E-positive malignancies, such as thyroid, lung, and colorectal cancers or hairy cell leukemia [182].

The TTM chelator inhibited the growth of melanoma cell lines resistant to BRAF or MEK1/2 inhibitors and increased the antineoplastic activity of these inhibitors [183]. In addition, in CRC cells carrying BRAF V600E mutations, Cu depletion induced by pharmacological treatment with TTM reduced the growth of BRAF V600E cells in colon cancers that were resistant to BRAF inhibitors [184]. Currently, this chelator is evaluated as an adjuvant therapy in various cancer clinical trials.

Bleomycin (a glycopeptidic antibiotic produced by *Streptomyces verticillus*) and curcumin (a phytochemical agent) are other chelators that gave promising results in oncology [185,186]. Bleomycin is regularly used in combination with other therapeutic agents such as cisplatin and etoposide in testicular cancer [187]. Curcumin may be used in monotherapy or in combination with other anticancer agents potentially for the prevention of cancer [188].

Copper ionophores are molecules that transport Cu ions through cellular membranes. Ionophores increase and/or redistribute intracellular Cu levels, often allowing Cu to become bioavailable [176]. These molecules have a high affinity for Cu(II) and a low affinity for Cu(I). With the cytosol of the cells being a reducing environment, the Cu entering the cell will be reduced into its Cu(I) oxidation form. Such a release of Cu(I) will poison the cell [189].

In the family of ionophores, several compounds such as docosahexaenoic acid (DHA), disulfiram (DSF), bis (thiosemicarbazone) copper complexes, and clioquinol can be found. The clinical use of clioquinol has been discontinued because of its neurotoxicity [190], but clioquinol or its analogues are still tested in combination or with different administration routes to maintain its anticancer effects while reducing toxicities [191]. The anticancer efficacy of DSF was demonstrated in in vitro and in vivo models of inflammatory breast cancer [192], and DSF is currently tested in clinical trials (clinicaltrials.gov id#: NCT04265274 and NCT03323346).

In addition, the combination of DSF and DHA has been shown to promote the death of cancer cells and to reduce the growth of cancer cells in vitro and in vivo [193]. One study has also suggested combining DSF with a PI3K inhibitor. This combination could be a new therapeutic strategy in breast cancer, particularly for patients with PIK3CA mutations [194]. In addition, coadministration of this drug with copper has shown inhibition of tumor growth in hormone-sensitive and castration-resistant models of the disease [195]. Finally, it has to be noticed that only the Cu-complexed form of these ionophores is active as a cancer treatment, i.e., disulfiram (DSF), bis (thiosemicarbazone) copper complexes, and clioquinol, because the ligands alone (metal-free compounds) have a minimal anticancer effect [196].

Some chelators such as curcumin or D-penicillamine penetrate cancer cells with difficulty because of their physicochemical properties. The development of innovative delivery systems for Cu-chelating agents should overcome these limitations and increase their efficacy and limit potential side effects [197,198,199]. Other strategies such as photochemical internalization (PCI) have been used to improve the intracellular delivery of bleomycin [200].

### 5.2. Copper-Based Nanoparticles and Metal-Based Strategies

Copper-based nanoparticles (CuNPs) have theranostic applications in oncology, i.e., they can be used for imaging or therapeutic purposes [201]. CuNPs can be used in a variety of therapeutic strategies, such as photothermal therapy combined with immunotherapies, to induce systemic immune responses against tumors [202]. The photothermal activity of other CuNPs was successfully exploited to induce the destruction of residual cancer cells and prevent local cancer recurrence in vivo after a single irradiation session [203]. The development of transferrin-based CuNPs loaded with doxorubicin successfully inhibited in vivo tumor growth [204].

A long-lasting active research effort has shown that copper-based radioisotopes have a promising future in the field of cancer diagnostics and therapeutics, especially for the ^64^Cu isotope [205,206]. In a model of human CRC in hamsters, ^64^Cu showed anticancer activity, and the survival was significantly increased [207]. Interestingly, the combination of the ^67^Cu radioisotope with an anti-L1-cell adhesion molecule monoclonal antibody reduced the growth of human metastatic ovarian cancer cells [208].

Metal-based therapies are major players in oncology. In this field, copper-based complexes have a promising future as presented previously. For this reason, the alteration of Cu metabolism in cancer is the basis for the development of copper complexes with antineoplastic characteristics [209,210].

### 5.3. Targeting Copper Metabolism Proteins

Finally, several therapeutic strategies either using or targeting Cu metabolism or mimicking Cu protein metabolism are currently investigated. Some of these strategies focus on the properties of SOD to develop a redox approach. One of the approaches aims at producing excess ROS by exploiting the properties of certain metals, which will lead to the death of cancer cells [211,212]. Opposite strategies that focus on the elimination of the toxic free radical and its derivatives via SOD or SOD mimicking compounds have also been developed [213]. The combination of this therapeutic strategy with radiotherapy or chemotherapy has shown promising results in preclinical trials. This strategy is just one example of the many approaches developed around superoxide dismutase [163]. Afterward, in view of the involvement of LOX family metalloenzyme in tumorigenesis and the formation of metastases, strategies specifically targeting proteins of the LOX family have been developed. During LOX studies, LOX propeptide has been shown to have an inhibitory effect in the development of cancerous tumors [214]. Different approaches explored to test the inhibition of LOX through the development of inhibitors of LOX isoforms such as recombinant LOX propeptides or via the use of therapeutic antibodies targeting LOX and LOXL2 [214,215].

## 6. Conclusions

Currently, the importance of copper in carcinogenesis and metastasis formation and in resistance to treatment has been explored. In addition, research work on copper deregulation in oncology and the recent understanding of copper metabolism have led to the development of numerous therapeutic strategies targeting this trace element. Despite numerous research studies and the improvement of knowledge on copper metabolism over the years, some shadow areas persist. Specific alterations in Cu metabolism seem to have a promising future in the clinic as prognostic and/or predictive biomarkers. In the coming years, copper and its metabolism will continue to play a significant role in both cancer diagnosis and therapeutic strategy.

## Figures and Tables

**Table 1:** The different Cu-binding proteins known in human cells. Inspired from Blockhuys, S et al. [3].

Cell Membrane	Intracellular Vesicles	Endoplasmic Reticulum	Cytoplasm	Extracellular Space	
**ATP7A**	TYR	MOXD1	Atox1	AOC1	SOD3
SLC31A1	TYRP1	MOXD2P	CCS	Cp	AFP
SLC31A2			COMMD1	DBH	ALB
AOC2	**Nucleus**	**Mitochondrion**	CUTC	ENOX1	F5
AOC3	COMMD1	COX11	LOXL3	ENOX2	GPC1
ENOX1	CUTC	COX17	MAP2K1	LOX	LTF
ENOX2	LOXL2	Sco1	MEMO1	LOXL1	MT3
HEPH	MAP2K1	Sco2	SOD1	LOXL2	MT4
HEPHL1	MEMO1	MT-CO1	MT3	LOXL3	S100A12/A13
PAM	PARK7	MT-CO2	PRNP	LOXL4	SNCA
PARK7	SOD1	PARK7	S100A12/A13	PAM	SPARC
APP	LTF	PRNP	SNCA		
GPC1	PRNP	CUTA		**Golgi apparatus**	
PRNP	S100A5		**Cytoskeleton**	ATP7A	
S100A12	S100B		MAP2K1	ATP7B	
SNCA	SNCA		S100A12	PRNP	

**Table 2:** Cu proteins affected in cancers and their prognostic relevance.

Altered Player	Regulation	Sample	Cancer	Prognostic	Ref.
Atox1	+	Tissue	Breast Invasive Cancer	poor	[3]
Atox1	+	Tissue	Melanoma	poor	[58]
Atox1	+	Tissue	Skin Cancer		[58]
Atox1	+		Blood		[58]
Atox1	+	Tissue	Colorectal Cancer	poor	[59]
ATP7A	+	Tissue	Pancreatic Cancer		[60]
ATP7A	+	Tissue	Breast Invasive Cancer	poor	[61]
ATP7A	+	Tissue/cell lines	Lung Cancer	poor	[62]
ATP7A	+	Tissue	Colorectal Cancer		[57,63]
ATP7A	+	Tissue/cell lines	Ovarian Cancer	poor	[64,65]
ATP7B	+	Tissue	Esophageal Carcinoma	poor	[66]
ATP7B	+	Tissue	Endometrial Carcinoma	poor	[67]
ATP7B	+	Tissue	Breast Invasive Cancer	poor	[68]
ATP7B	+	Tissue	Oral Squamous Cell Carcinoma	poor	[69]
ATP7B	+	Tissue	Gastric Carcinoma	poor	[70]
ATP7B	+	Tissue	Hepatocellular Carcinoma	poor	[71]
LOX-1	+	Tissue	Pancreatic Cancer	poor	[72]
LOX-1	+	Tissue	Prostate Cancer	poor	[73]
LOX	+	Tissue	Colorectal Cancer	poor	[74]
LOX-2	+	Tissue/Serum	Hepatocellular Carcinoma	poor	[75]
LOX	+	Tissue	Breast Invasive Cancer	poor	[76,77,78]
LOX	−	Tissue	Bronchogenic Carcinoma	poor	[79]
LOX	−	Tissue/cell lines	Gastric Cancers		[80]
LOX	+	Tissue	Head and Neck Squamous Cell Carcinoma	poor	[81]
LOX	+	Tissue/cell lines	Lung Adenocarcinoma	poor	[82,83]
LOX	+	Cell lines	Melanoma	poor	[76]
LOX	+	Tissue	Oral and Oropharyngeal Squamous Cell	poor	[84]
LOX	−	Tissue/cell lines	Basal and Squamous Cell Carcinomas		[85]
LOX	+	Tissue	Renal Cell Carcinoma		[86]
LOXL1	−	Cell lines	Bladder Cancer		[87]
LOXL4	−	Cell lines	Bladder Cancer		[87]
LOXL1	+	Cell lines	Lung Adenocarcinoma		[88]
LOXL1	+	Tissue	Salivary Gland Adenoid Cystic Carcinoma		[89]
LOXL2	+	Tissue/cell lines	Breast Invasive Cancer	poor	[90,91,92,93,94]
LOXL2	+	Tissue	Colorectal Cancer	poor	[93,95,96,97]
LOXL2	+	Tissue/cell lines	Gastric Cancers	poor	[93,98]
LOXL2	+	Tissue	Endometrial Cancer	poor	[94]
LOXL2	+	Tissue	Testicular Cancer	poor	[94]
LOXL2	+	Tissue	Hepatocellular Carcinoma	poor	[94]
LOXL2	+	Tissue	Lung Squamous Cell Carcinoma	poor	[90,99]
LOXL2	+	Cell lines	Melanoma	poor	[76]
LOXL2	+	Tissue/cell lines	Pancreatic Cancer	poor	[94,100]
LOXL2	+	Tissue	Prostate Cancer	poor	[101]
LOXL2	+	Tissue	Renal Cell Carcinoma	poor	[94]
LOXL2	+	Tissue	Laryngeal Cancer	poor	[90,94]
LOXL2	+	Tissue	Esophageal Squamous Cell	poor	[94,102]
LOXL3	+	Cell lines	Breast Invasive Cancer		[103]
LOXL3	+	Cell lines	Melanoma		[76,103]
LOXL4	+	Tissue	Colorectal Adenocarcinoma		[95]
LOXL4	+	Tissue/cell lines	Head and Neck Squamous Cell Carcinoma		[104,105,106]
Ctr1	+	Tissue	Colorectal Cancer		[57]
Sco1	+	Tissue	Colorectal Cancer		[57]
COX11	+	Tissue	Colorectal Cancer		[57]
Ctr1	+	Tissue	Non-Small-Cell Lung Cancer	good	[107]
Ctr1	Variable	Tissue	Ovarian Cancer		[108]
Ctr2	Variable	Tissue	Ovarian Cancer		[108]
Ctr1	+	Cell lines	Ovarian Cancer		[109]
Ctr1	+	Tissue	Breast Invasive Cancer		[3]
Cu	+	Serum	Bladder Cancer		[40]
Zn	−	Serum	Bladder Cancer		[40]
Cu	+	Serum	Breast Invasive Cancer		[42,44,49,110]
Cu/Zn	+	Serum	Breast Invasive Cancer		[42,44,110]
Cu	+	Serum/Tissue	Lung Cancer		[51,53]
Cu/Zn	+	Serum	Lung Cancer		[51]
Cu	±	Serum/Tissue	Colorectal Cancer		[41,45,48]
Cu/Zn	+	Serum/Tissue	Colorectal Cancer		[41,45,48]
Cu/Zn	+	Serum	Pancreatic Cancer		[46]
Cu	−	Serum	Pancreatic Cancer		[46]
Cu	+	Serum	Cervical Cancer and Uterine Myoma		[47]
Cu/Zn	+	Serum	Cervical Cancer and Uterine Myoma		[47]
Cu	+	Serum	Prostate Cancer		[43]
Cu	+	Serum	Papillary Thyroid Carcinoma		[111]
Cu	−	Serum	Endometrial Cancer		[112]
GSH	+	Tissue	Hepatocellular Carcinoma		[113]
CCS	+	Tissue	Brain Cancer		[58]
CCS	+	Tissue	Ovarian Cancer		[58]
CCS	−	Tissue	Hepatocellular Carcinoma		[58]
CCS	−	Tissue	Prostate Cancer		[58]
SOD3	−	Tissue	Lung Cancer	poor	[114,115,116]
SOD3	−	Tissue	Breast Invasive Cancer	poor	[117,118]
SOD3	−	Tissue	Prostate Cancer	poor	[119,120]
SOD3	−	Tissue	Pancreatic Cancer	poor	[121]
SOD3	−	Tissue	Colorectal Cancer		[122]
SOD3	−	Cell lines	Thyroid Cancer		[123]
SOD3	+	Serum	Gastric Adenocarcinoma		[124]
COX17	+	Cell lines	Lung Cancer		[125]
COX17	+	Tissue	Head and Neck Squamous Cell Carcinomas		[126]
